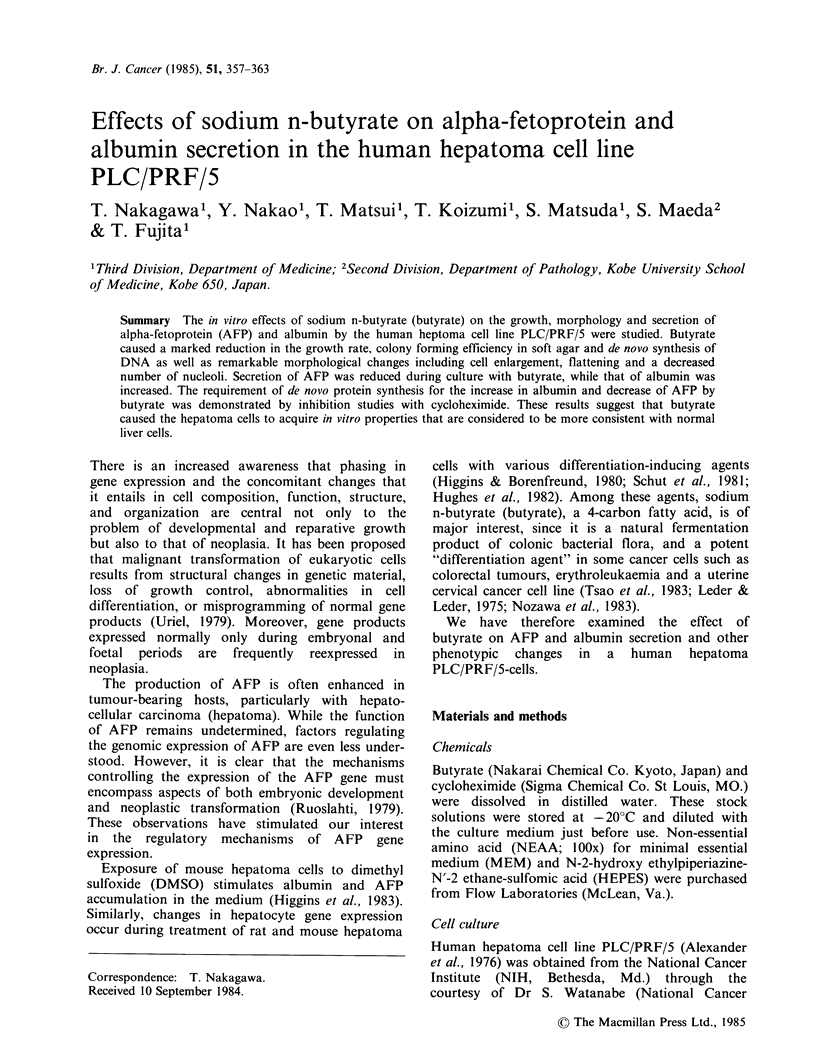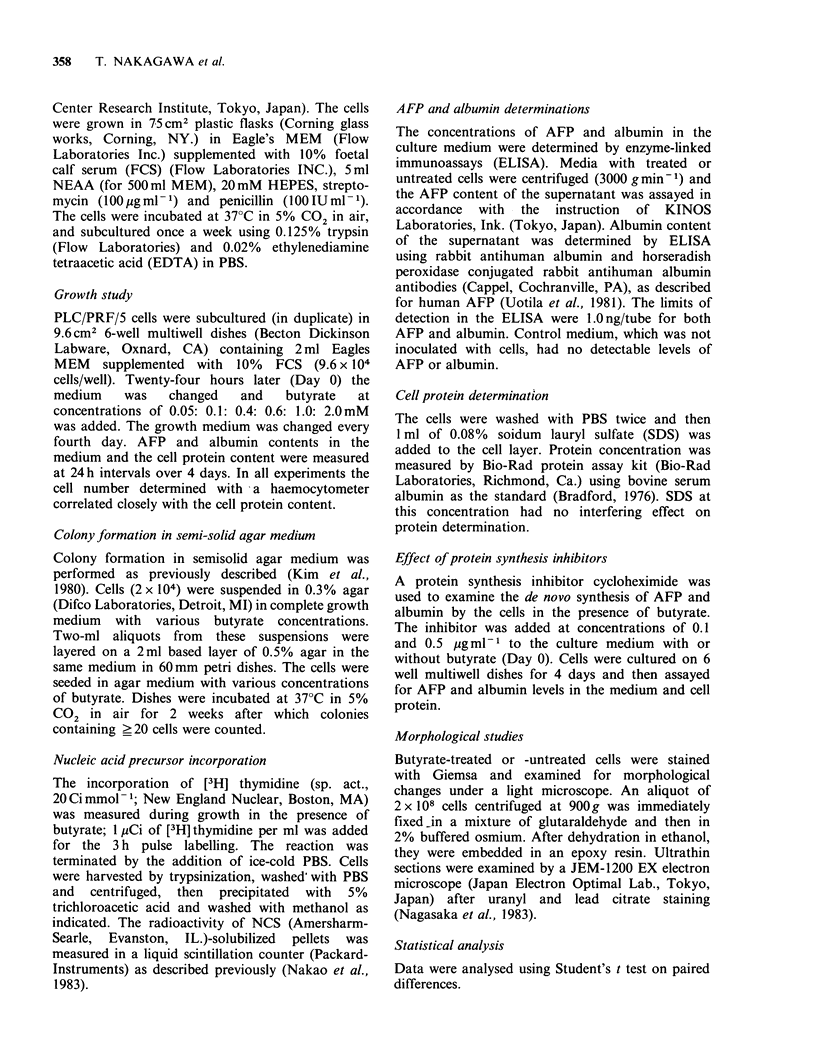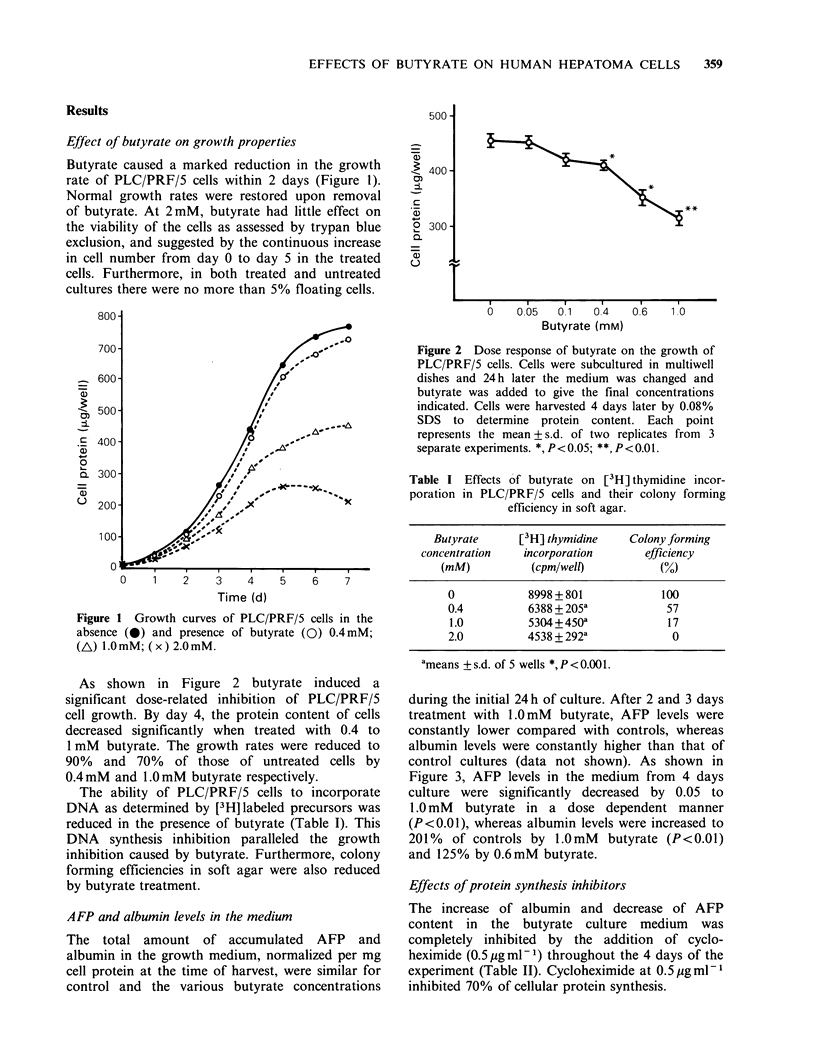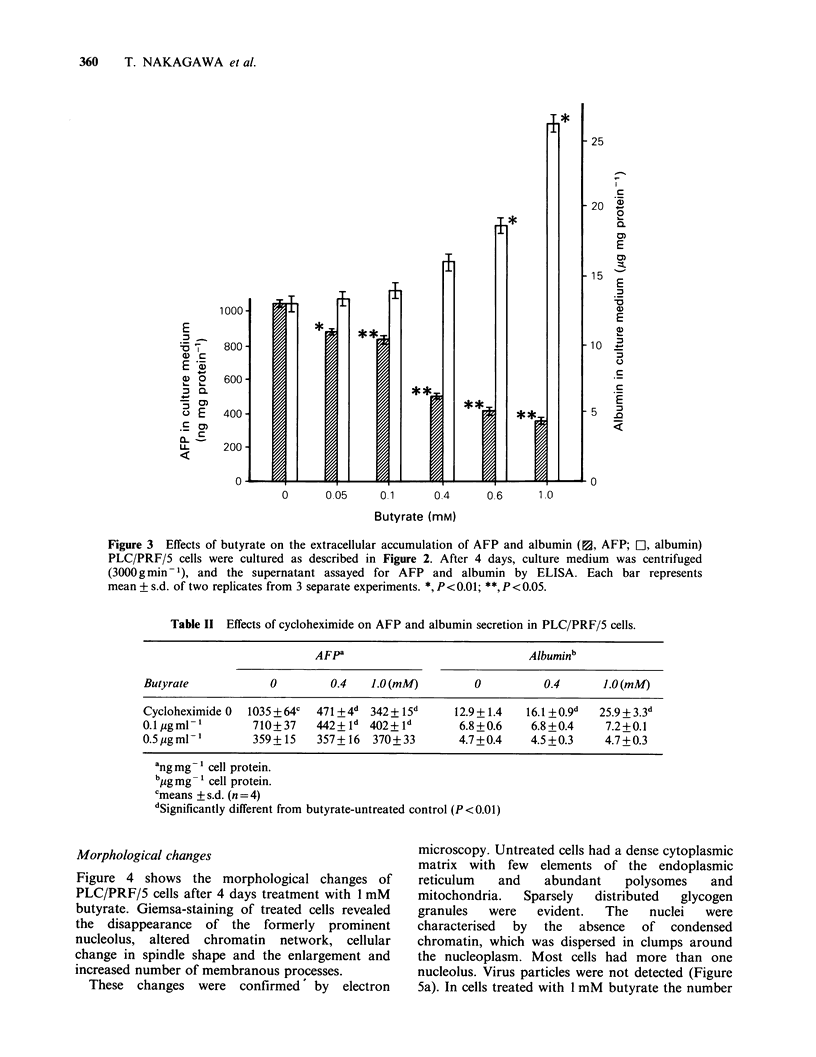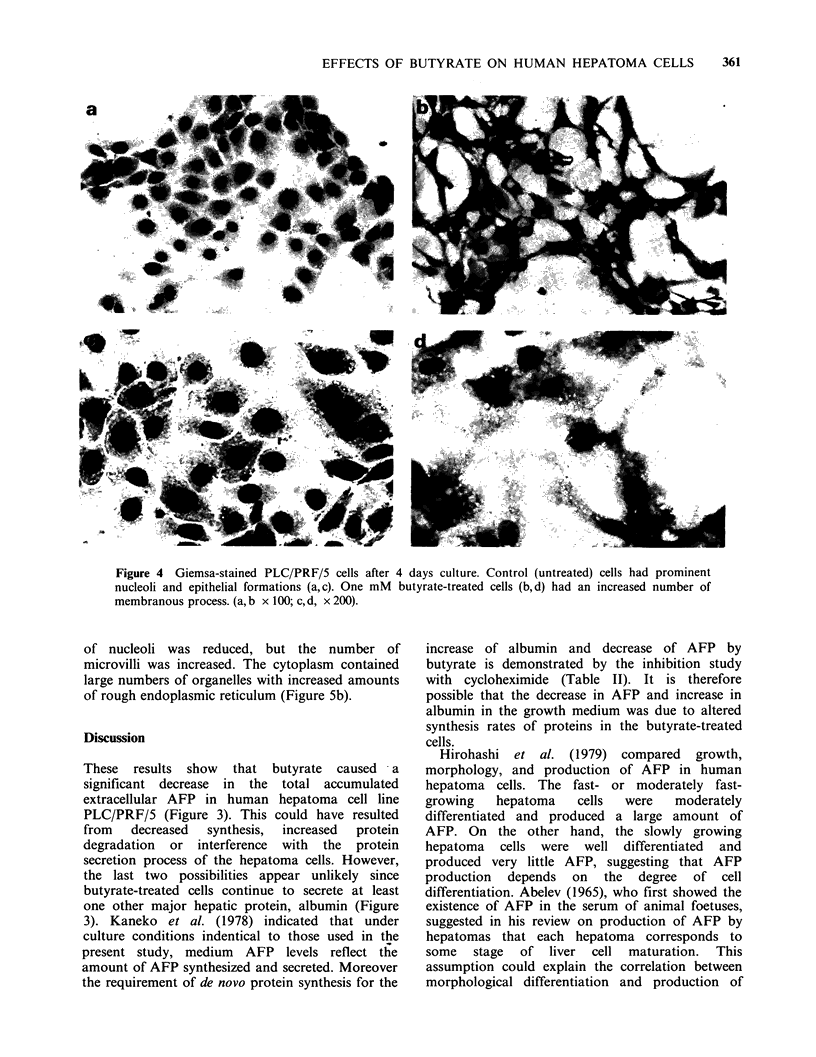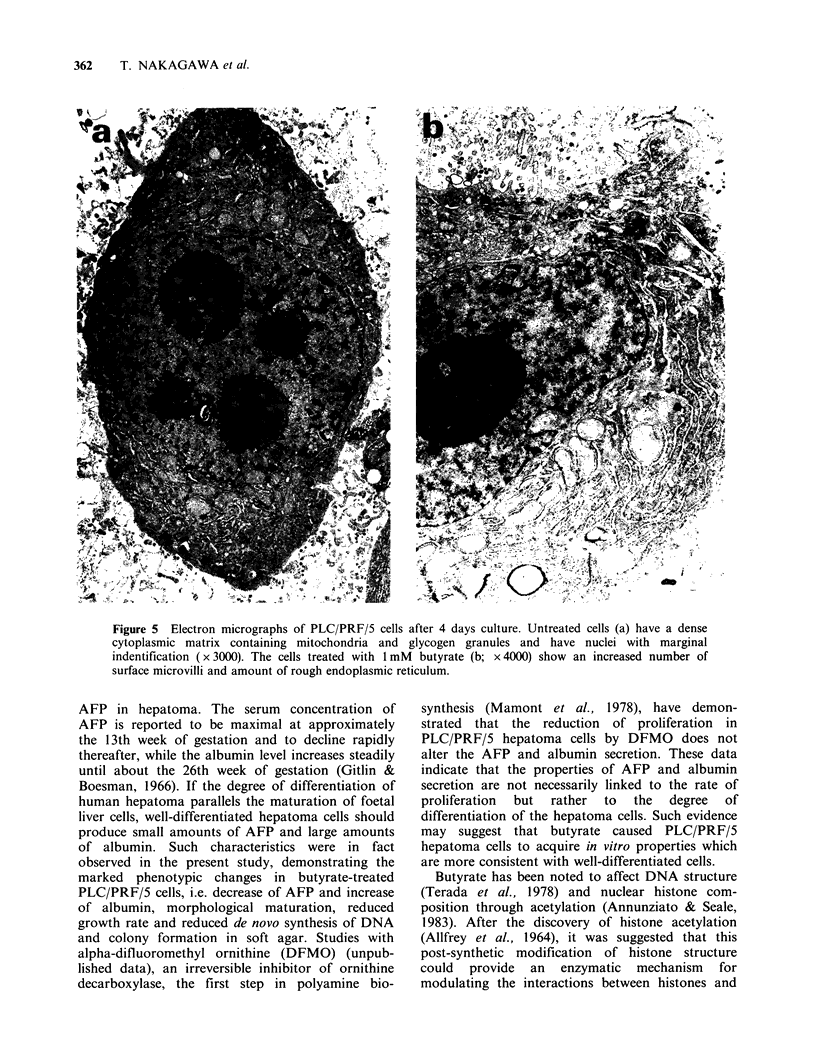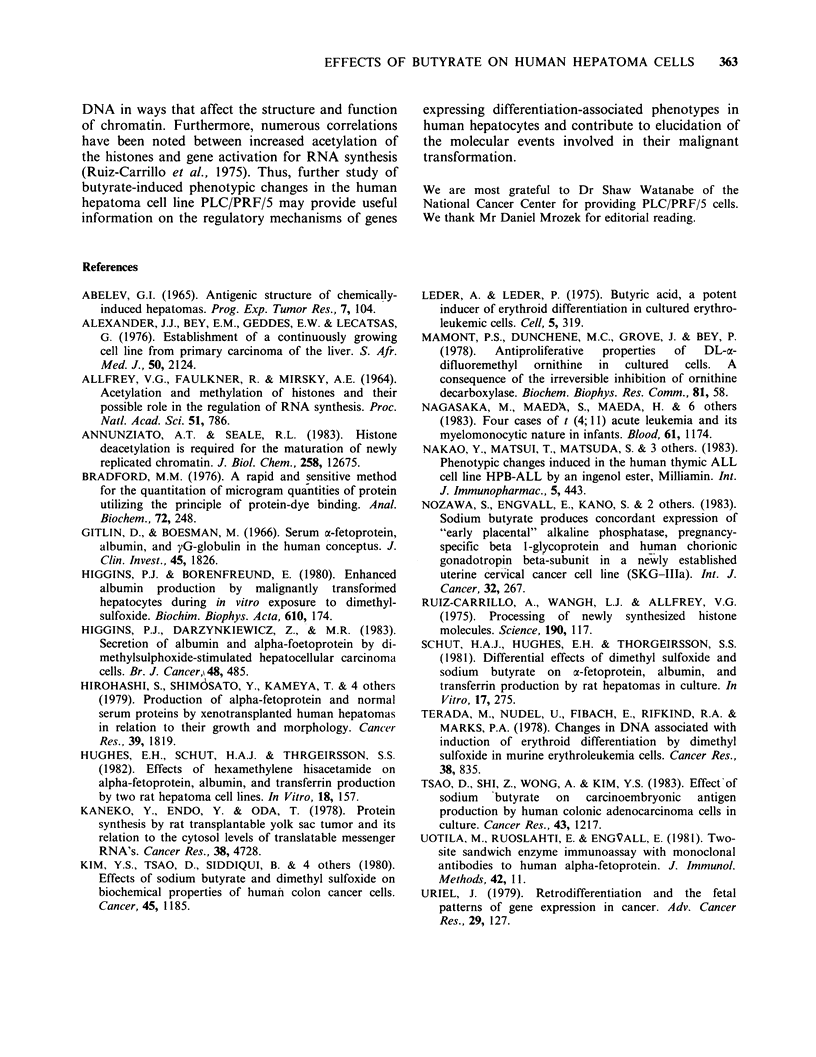# Effects of sodium n-butyrate on alpha-fetoprotein and albumin secretion in the human hepatoma cell line PLC/PRF/5.

**DOI:** 10.1038/bjc.1985.47

**Published:** 1985-03

**Authors:** T. Nakagawa, Y. Nakao, T. Matsui, T. Koizumi, S. Matsuda, S. Maeda, T. Fujita

## Abstract

**Images:**


Br. J. Cancer (1985), **51**, 357–363

### Effects of sodium n-butyrate on alpha-fetoprotein and albumin secretion in the human hepatoma cell line PLC/PRF/5

T. Nakagawa[1], Y. Nakao[1], T. Matsui[1], T. Koizumi[1], S. Matsuda[1], S. Maeda[2] & T. Fujita[1]

[1]*Third Division, Department of Medicine;* [2]*Second Division, Department of Pathology, Kobe University School of Medicine, Kobe 650, Japan.*

**Summary** The *in vitro* effects of sodium n-butyrate (butyrate) on the growth, morphology and secretion of alpha-fetoprotein (AFP) and albumin by the human heptoma cell line PLC/PRF/5 were studied. Butyrate caused a marked reduction in the growth rate, colony forming efficiency in soft agar and *de novo* synthesis of DNA as well as remarkable morphological changes including cell enlargement, flattening and a decreased number of nucleoli. Secretion of AFP was reduced during culture with butyrate, while that of albumin was increased. The requirement of *de novo* protein synthesis for the increase in albumin and decrease of AFP by butyrate was demonstrated by inhibition studies with cycloheximide. These results suggest that butyrate caused the hepatoma cells to acquire *in vitro* properties that are considered to be more consistent with normal liver cells.

There is an increased awareness that phasing in gene expression and the concomitant changes that it entails in cell composition, function, structure, and organization are central not only to the problem of developmental and reparative growth but also to that of neoplasia. It has been proposed that malignant transformation of eukaryotic cells results from structural changes in genetic material, loss of growth control, abnormalities in cell differentiation, or misprogramming of normal gene products (Uriel, 1979). Moreover, gene products expressed normally only during embryonal and foetal periods are frequently reexpressed in neoplasia.

The production of AFP is often enhanced in tumour-bearing hosts, particularly with hepato-cellular carcinoma (hepatoma). While the function of AFP remains undetermined, factors regulating the genomic expression of AFP are even less under-stood. However, it is clear that the mechanisms controlling the expression of the AFP gene must encompass aspects of both embryonic development and neoplastic transformation (Ruoslahti, 1979). These observations have stimulated our interest in the regulatory mechanisms of AFP gene expression.

Exposure of mouse hepatoma cells to dimethyl sulfoxide (DMSO) stimulates albumin and AFP accumulation in the medium (Higgins *et al.*, 1983). Similarly, changes in hepatocyte gene expression occur during treatment of rat and mouse hepatoma cells with various differentiation-inducing agents (Higgins & Borenfreund, 1980; Schut *et al.*, 1981; Hughes *et al.*, 1982). Among these agents, sodium n-butyrate (butyrate), a 4-carbon fatty acid, is of major interest, since it is a natural fermentation product of colonic bacterial flora, and a potent "differentiation agent" in some cancer cells such as colorectal tumours, erythroleukaemia and a uterine cervical cancer cell line (Tsao *et al.*, 1983; Leder & Leder, 1975; Nozawa *et al.*, 1983).

We have therefore examined the effect of butyrate on AFP and albumin secretion and other phenotypic changes in a human hepatoma PLC/PRF/5-cells.

### Materials and methods

### *Chemicals*

Butyrate (Nakarai Chemical Co. Kyoto, Japan) and cycloheximide (Sigma Chemical Co. St Louis, MO.) were dissolved in distilled water. These stock solutions were stored at $-20°C$ and diluted with the culture medium just before use. Non-essential amino acid (NEAA; 100x) for minimal essential medium (MEM) and N-2-hydroxy ethylpiperiazine-N′-2 ethane-sulfomic acid (HEPES) were purchased from Flow Laboratories (McLean, Va.).

### *Cell culture*

Human hepatoma cell line PLC/PRF/5 (Alexander *et al.*, 1976) was obtained from the National Cancer Institute (NIH, Bethesda, Md.) through the courtesy of Dr S. Watanabe (National Cancer

Correspondence: T. Nakagawa.
Received 10 September 1984.

© The Macmillan Press Ltd., 1985

Center Research Institute, Tokyo, Japan). The cells were grown in 75 $cm^2$ plastic flasks (Corning glass works, Corning, NY.) in Eagle's MEM (Flow Laboratories Inc.) supplemented with 10% foetal calf serum (FCS) (Flow Laboratories INC.), 5 ml NEAA (for 500 ml MEM), 20 mM HEPES, streptomycin (100 $\mu g\, ml^{-1}$) and penicillin (100 IU $ml^{-1}$). The cells were incubated at 37°C in 5% $CO_2$ in air, and subcultured once a week using 0.125% trypsin (Flow Laboratories) and 0.02% ethylenediamine tetraacetic acid (EDTA) in PBS.

*Growth study*

PLC/PRF/5 cells were subcultured (in duplicate) in 9.6 $cm^2$ 6-well multiwell dishes (Becton Dickinson Labware, Oxnard, CA) containing 2 ml Eagles MEM supplemented with 10% FCS ($9.6 \times 10^4$ cells/well). Twenty-four hours later (Day 0) the medium was changed and butyrate at concentrations of 0.05: 0.1: 0.4: 0.6: 1.0: 2.0 mM was added. The growth medium was changed every fourth day. AFP and albumin contents in the medium and the cell protein content were measured at 24 h intervals over 4 days. In all experiments the cell number determined with a haemocytometer correlated closely with the cell protein content.

*Colony formation in semi-solid agar medium*

Colony formation in semisolid agar medium was performed as previously described (Kim *et al.*, 1980). Cells ($2 \times 10^4$) were suspended in 0.3% agar (Difco Laboratories, Detroit, MI) in complete growth medium with various butyrate concentrations. Two-ml aliquots from these suspensions were layered on a 2 ml based layer of 0.5% agar in the same medium in 60 mm petri dishes. The cells were seeded in agar medium with various concentrations of butyrate. Dishes were incubated at 37°C in 5% $CO_2$ in air for 2 weeks after which colonies containing $\geqq 20$ cells were counted.

*Nucleic acid precursor incorporation*

The incorporation of [$^3$H] thymidine (sp. act., 20 Ci $mmol^{-1}$; New England Nuclear, Boston, MA) was measured during growth in the presence of butyrate; 1 $\mu$Ci of [$^3$H] thymidine per ml was added for the 3 h pulse labelling. The reaction was terminated by the addition of ice-cold PBS. Cells were harvested by trypsinization, washed with PBS and centrifuged, then precipitated with 5% trichloroacetic acid and washed with methanol as indicated. The radioactivity of NCS (Amersharm-Searle, Evanston, IL.)-solubilized pellets was measured in a liquid scintillation counter (Packard-Instruments) as described previously (Nakao *et al.*, 1983).

*AFP and albumin determinations*

The concentrations of AFP and albumin in the culture medium were determined by enzyme-linked immunoassays (ELISA). Media with treated or untreated cells were centrifuged (3000 $g\, min^{-1}$) and the AFP content of the supernatant was assayed in accordance with the instruction of KINOS Laboratories, Ink. (Tokyo, Japan). Albumin content of the supernatant was determined by ELISA using rabbit antihuman albumin and horseradish peroxidase conjugated rabbit antihuman albumin antibodies (Cappel, Cochranville, PA), as described for human AFP (Uotila *et al.*, 1981). The limits of detection in the ELISA were 1.0 ng/tube for both AFP and albumin. Control medium, which was not inoculated with cells, had no detectable levels of AFP or albumin.

*Cell protein determination*

The cells were washed with PBS twice and then 1 ml of 0.08% soidum lauryl sulfate (SDS) was added to the cell layer. Protein concentration was measured by Bio-Rad protein assay kit (Bio-Rad Laboratories, Richmond, Ca.) using bovine serum albumin as the standard (Bradford, 1976). SDS at this concentration had no interfering effect on protein determination.

*Effect of protein synthesis inhibitors*

A protein synthesis inhibitor cycloheximide was used to examine the *de novo* synthesis of AFP and albumin by the cells in the presence of butyrate. The inhibitor was added at concentrations of 0.1 and 0.5 $\mu g\, ml^{-1}$ to the culture medium with or without butyrate (Day 0). Cells were cultured on 6 well multiwell dishes for 4 days and then assayed for AFP and albumin levels in the medium and cell protein.

*Morphological studies*

Butyrate-treated or -untreated cells were stained with Giemsa and examined for morphological changes under a light microscope. An aliquot of $2 \times 10^8$ cells centrifuged at 900 *g* was immediately fixed in a mixture of glutaraldehyde and then in 2% buffered osmium. After dehydration in ethanol, they were embedded in an epoxy resin. Ultrathin sections were examined by a JEM-1200 EX electron microscope (Japan Electron Optimal Lab., Tokyo, Japan) after uranyl and lead citrate staining (Nagasaka *et al.*, 1983).

*Statistical analysis*

Data were analysed using Student's *t* test on paired differences.

### Results

### *Effect of butyrate on growth properties*

Butyrate caused a marked reduction in the growth rate of PLC/PRF/5 cells within 2 days (Figure 1). Normal growth rates were restored upon removal of butyrate. At 2 mM, butyrate had little effect on the viability of the cells as assessed by trypan blue exclusion, and suggested by the continuous increase in cell number from day 0 to day 5 in the treated cells. Furthermore, in both treated and untreated cultures there were no more than 5% floating cells.

**Figure 1** Growth curves of PLC/PRF/5 cells in the absence (●) and presence of butyrate (○) 0.4 mM; (△) 1.0 mM; (×) 2.0 mM.

As shown in Figure 2 butyrate induced a significant dose-related inhibition of PLC/PRF/5 cell growth. By day 4, the protein content of cells decreased significantly when treated with 0.4 to 1 mM butyrate. The growth rates were reduced to 90% and 70% of those of untreated cells by 0.4 mM and 1.0 mM butyrate respectively.

The ability of PLC/PRF/5 cells to incorporate DNA as determined by [$^3$H]labeled precursors was reduced in the presence of butyrate (Table I). This DNA synthesis inhibition paralleled the growth inhibition caused by butyrate. Furthermore, colony forming efficiencies in soft agar were also reduced by butyrate treatment.

### *AFP and albumin levels in the medium*

The total amount of accumulated AFP and albumin in the growth medium, normalized per mg cell protein at the time of harvest, were similar for control and the various butyrate concentrations

**Figure 2** Dose response of butyrate on the growth of PLC/PRF/5 cells. Cells were subcultured in multiwell dishes and 24 h later the medium was changed and butyrate was added to give the final concentrations indicated. Cells were harvested 4 days later by 0.08% SDS to determine protein content. Each point represents the mean ± s.d. of two replicates from 3 separate experiments. *, $P<0.05$; **, $P<0.01$.

**Table I** Effects of butyrate on [$^3$H]thymidine incorporation in PLC/PRF/5 cells and their colony forming efficiency in soft agar.

| *Butyrate concentration (mM)* | *[$^3$H] thymidine incorporation (cpm/well)* | *Colony forming efficiency (%)* |
|---|---|---|
| 0 | 8998 ± 801 | 100 |
| 0.4 | 6388 ± 205[a] | 57 |
| 1.0 | 5304 ± 450[a] | 17 |
| 2.0 | 4538 ± 292[a] | 0 |

[a]means ± s.d. of 5 wells *, $P<0.001$.

during the initial 24 h of culture. After 2 and 3 days treatment with 1.0 mM butyrate, AFP levels were constantly lower compared with controls, whereas albumin levels were constantly higher than that of control cultures (data not shown). As shown in Figure 3, AFP levels in the medium from 4 days culture were significantly decreased by 0.05 to 1.0 mM butyrate in a dose dependent manner ($P<0.01$), whereas albumin levels were increased to 201% of controls by 1.0 mM butyrate ($P<0.01$) and 125% by 0.6 mM butyrate.

### *Effects of protein synthesis inhibitors*

The increase of albumin and decrease of AFP content in the butyrate culture medium was completely inhibited by the addition of cycloheximide (0.5 $\mu g\,ml^{-1}$) throughout the 4 days of the experiment (Table II). Cycloheximide at 0.5 $\mu g\,ml^{-1}$ inhibited 70% of cellular protein synthesis.

**Figure 3** Effects of butyrate on the extracellular accumulation of AFP and albumin (▨, AFP; □, albumin) PLC/PRF/5 cells were cultured as described in **Figure 2**. After 4 days, culture medium was centrifuged ($3000\,g\,min^{-1}$), and the supernatant assayed for AFP and albumin by ELISA. Each bar represents mean ± s.d. of two replicates from 3 separate experiments. *, $P<0.01$; **, $P<0.05$.

**Table II** Effects of cycloheximide on AFP and albumin secretion in PLC/PRF/5 cells.

| | *AFP*[a] | | | *Albumin*[b] | | |
|---|---|---|---|---|---|---|
| *Butyrate* | *0* | *0.4* | *1.0 (mM)* | *0* | *0.4* | *1.0 (mM)* |
| Cycloheximide 0 | 1035±64[c] | 471±4[d] | 342±15[d] | 12.9±1.4 | 16.1±0.9[d] | 25.9±3.3[d] |
| 0.1 $\mu g\,ml^{-1}$ | 710±37 | 442±1[d] | 402±1[d] | 6.8±0.6 | 6.8±0.4 | 7.2±0.1 |
| 0.5 $\mu g\,ml^{-1}$ | 359±15 | 357±16 | 370±33 | 4.7±0.4 | 4.5±0.3 | 4.7±0.3 |

[a]$ng\,mg^{-1}$ cell protein.
[b]$\mu g\,mg^{-1}$ cell protein.
[c]means ±s.d. ($n=4$)
[d]Significantly different from butyrate-untreated control ($P<0.01$)

*Morphological changes*

Figure 4 shows the morphological changes of PLC/PRF/5 cells after 4 days treatment with 1 mM butyrate. Giemsa-staining of treated cells revealed the disappearance of the formerly prominent nucleolus, altered chromatin network, cellular change in spindle shape and the enlargement and increased number of membranous processes.

These changes were confirmed by electron microscopy. Untreated cells had a dense cytoplasmic matrix with few elements of the endoplasmic reticulum and abundant polysomes and mitochondria. Sparsely distributed glycogen granules were evident. The nuclei were characterised by the absence of condensed chromatin, which was dispersed in clumps around the nucleoplasm. Most cells had more than one nucleolus. Virus particles were not detected (Figure 5a). In cells treated with 1 mM butyrate the number

**Figure 4** Giemsa-stained PLC/PRF/5 cells after 4 days culture. Control (untreated) cells had prominent nucleoli and epithelial formations (a, c). One mM butyrate-treated cells (b, d) had an increased number of membranous process. (a, b × 100; c, d, × 200).

of nucleoli was reduced, but the number of microvilli was increased. The cytoplasm contained large numbers of organelles with increased amounts of rough endoplasmic reticulum (Figure 5b).

### Discussion

These results show that butyrate caused a significant decrease in the total accumulated extracellular AFP in human hepatoma cell line PLC/PRF/5 (Figure 3). This could have resulted from decreased synthesis, increased protein degradation or interference with the protein secretion process of the hepatoma cells. However, the last two possibilities appear unlikely since butyrate-treated cells continue to secrete at least one other major hepatic protein, albumin (Figure 3). Kaneko *et al.* (1978) indicated that under culture conditions indentical to those used in the present study, medium AFP levels reflect the amount of AFP synthesized and secreted. Moreover the requirement of *de novo* protein synthesis for the increase of albumin and decrease of AFP by butyrate is demonstrated by the inhibition study with cycloheximide (Table II). It is therefore possible that the decrease in AFP and increase in albumin in the growth medium was due to altered synthesis rates of proteins in the butyrate-treated cells.

Hirohashi *et al.* (1979) compared growth, morphology, and production of AFP in human hepatoma cells. The fast- or moderately fast-growing hepatoma cells were moderately differentiated and produced a large amount of AFP. On the other hand, the slowly growing hepatoma cells were well differentiated and produced very little AFP, suggesting that AFP production depends on the degree of cell differentiation. Abelev (1965), who first showed the existence of AFP in the serum of animal foetuses, suggested in his review on production of AFP by hepatomas that each hepatoma corresponds to some stage of liver cell maturation. This assumption could explain the correlation between morphological differentiation and production of

**Figure 5** Electron micrographs of PLC/PRF/5 cells after 4 days culture. Untreated cells (a) have a dense cytoplasmic matrix containing mitochondria and glycogen granules and have nuclei with marginal indentification (×3000). The cells treated with 1 mM butyrate (b; ×4000) show an increased number of surface microvilli and amount of rough endoplasmic reticulum.

AFP in hepatoma. The serum concentration of AFP is reported to be maximal at approximately the 13th week of gestation and to decline rapidly thereafter, while the albumin level increases steadily until about the 26th week of gestation (Gitlin & Boesman, 1966). If the degree of differentiation of human hepatoma parallels the maturation of foetal liver cells, well-differentiated hepatoma cells should produce small amounts of AFP and large amounts of albumin. Such characteristics were in fact observed in the present study, demonstrating the marked phenotypic changes in butyrate-treated PLC/PRF/5 cells, i.e. decrease of AFP and increase of albumin, morphological maturation, reduced growth rate and reduced *de novo* synthesis of DNA and colony formation in soft agar. Studies with alpha-difluoromethyl ornithine (DFMO) (unpublished data), an irreversible inhibitor of ornithine decarboxylase, the first step in polyamine biosynthesis (Mamont *et al.,* 1978), have demonstrated that the reduction of proliferation in PLC/PRF/5 hepatoma cells by DFMO does not alter the AFP and albumin secretion. These data indicate that the properties of AFP and albumin secretion are not necessarily linked to the rate of proliferation but rather to the degree of differentiation of the hepatoma cells. Such evidence may suggest that butyrate caused PLC/PRF/5 hepatoma cells to acquire *in vitro* properties which are more consistent with well-differentiated cells.

Butyrate has been noted to affect DNA structure (Terada *et al.,* 1978) and nuclear histone composition through acetylation (Annunziato & Seale, 1983). After the discovery of histone acetylation (Allfrey *et al.,* 1964), it was suggested that this post-synthetic modification of histone structure could provide an enzymatic mechanism for modulating the interactions between histones and

DNA in ways that affect the structure and function of chromatin. Furthermore, numerous correlations have been noted between increased acetylation of the histones and gene activation for RNA synthesis (Ruiz-Carrillo *et al.*, 1975). Thus, further study of butyrate-induced phenotypic changes in the human hepatoma cell line PLC/PRF/5 may provide useful information on the regulatory mechanisms of genes expressing differentiation-associated phenotypes in human hepatocytes and contribute to elucidation of the molecular events involved in their malignant transformation.

We are most grateful to Dr Shaw Watanabe of the National Cancer Center for providing PLC/PRF/5 cells. We thank Mr Daniel Mrozek for editorial reading.

### References

ABELEV, G.I. (1965). Antigenic structure of chemically-induced hepatomas. *Prog. Exp. Tumor Res.*, **7**, 104.

ALEXANDER, J.J., BEY, E.M., GEDDES, E.W. & LECATSAS, G. (1976). Establishment of a continuously growing cell line from primary carcinoma of the liver. *S. Afr. Med. J.*, **50**, 2124.

ALLFREY, V.G., FAULKNER, R. & MIRSKY, A.E. (1964). Acetylation and methylation of histones and their possible role in the regulation of RNA synthesis. *Proc. Natl. Acad. Sci.* **51**, 786.

ANNUNZIATO, A.T. & SEALE, R.L. (1983). Histone deacetylation is required for the maturation of newly replicated chromatin. *J. Biol. Chem.*, **258**, 12675.

BRADFORD, M.M. (1976). A rapid and sensitive method for the quantitation of microgram quantities of protein utilizing the principle of protein-dye binding. *Anal. Biochem.*, **72**, 248.

GITLIN, D., & BOESMAN, M. (1966). Serum α-fetoprotein, albumin, and γG-globulin in the human conceptus. *J. Clin. Invest.*, **45**, 1826.

HIGGINS, P.J. & BORENFREUND, E. (1980). Enhanced albumin production by malignantly transformed hepatocytes during *in vitro* exposure to dimethylsulfoxide. *Biochim. Biophys. Acta*, **610**, 174.

HIGGINS, P.J., DARZYNKIEWICZ, Z., & M.R. (1983). Secretion of albumin and alpha-foetoprotein by dimethylsulphoxide-stimulated hepatocellular carcinoma cells. *Br. J. Cancer*, **48**, 485.

HIROHASHI, S., SHIMOSATO, Y., KAMEYA, T. & 4 others (1979). Production of alpha-fetoprotein and normal serum proteins by xenotransplanted human hepatomas in relation to their growth and morphology. *Cancer Res.*, **39**, 1819.

HUGHES, E.H., SCHUT, H.A.J. & THRGEIRSSON, S.S. (1982). Effects of hexamethylene hisacetamide on alpha-fetoprotein, albumin, and transferrin production by two rat hepatoma cell lines. *In Vitro*, **18**, 157.

KANEKO, Y., ENDO, Y. & ODA, T. (1978). Protein synthesis by rat transplantable yolk sac tumor and its relation to the cytosol levels of translatable messenger RNA's. *Cancer Res.*, **38**, 4728.

KIM, Y.S., TSAO, D., SIDDIQUI, B. & 4 others (1980). Effects of sodium butyrate and dimethyl sulfoxide on biochemical properties of human colon cancer cells. *Cancer*, **45**, 1185.

LEDER, A. & LEDER, P. (1975). Butyric acid, a potent inducer of erythroid differentiation in cultured erythroleukemic cells. *Cell*, **5**, 319.

MAMONT, P.S., DUNCHENE, M.C., GROVE, J. & BEY, P. (1978). Antiproliferative properties of DL-α-difluoremethyl ornithine in cultured cells. A consequence of the irreversible inhibition of ornithine decarboxylase. *Biochem. Biophys. Res. Comm.*, **81**, 58.

NAGASAKA, M., MAEDA, S., MAEDA, H. & 6 others (1983). Four cases of *t* (4;11) acute leukemia and its myelomonocytic nature in infants. *Blood*, **61**, 1174.

NAKAO, Y., MATSUI, T., MATSUDA, S. & 3 others. (1983). Phenotypic changes induced in the human thymic ALL cell line HPB-ALL by an ingenol ester, Milliamin. *Int. J. Immunopharmac.*, **5**, 443.

NOZAWA, S., ENGVALL, E., KANO, S. & 2 others. (1983). Sodium butyrate produces concordant expression of "early placental" alkaline phosphatase, pregnancy-specific beta 1-glycoprotein and human chorionic gonadotropin beta-subunit in a newly established uterine cervical cancer cell line (SKG–IIIa). *Int. J. Cancer*, **32**, 267.

RUIZ-CARRILLO, A., WANGH, L.J. & ALLFREY, V.G. (1975). Processing of newly synthesized histone molecules. *Science*, **190**, 117.

SCHUT, H.A.J., HUGHES, E.H. & THORGEIRSSON, S.S. (1981). Differential effects of dimethyl sulfoxide and sodium butyrate on α-fetoprotein, albumin, and transferrin production by rat hepatomas in culture. *In Vitro*, **17**, 275.

TERADA, M., NUDEL, U., FIBACH, E., RIFKIND, R.A. & MARKS, P.A. (1978). Changes in DNA associated with induction of erythroid differentiation by dimethyl sulfoxide in murine erythroleukemia cells. *Cancer Res.*, **38**, 835.

TSAO, D., SHI, Z., WONG, A. & KIM, Y.S. (1983). Effect of sodium butyrate on carcinoembryonic antigen production by human colonic adenocarcinoma cells in culture. *Cancer Res.*, **43**, 1217.

UOTILA, M., RUOSLAHTI, E. & ENGVALL, E. (1981). Two-site sandwich enzyme immunoassay with monoclonal antibodies to human alpha-fetoprotein. *J. Immunol. Methods*, **42**, 11.

URIEL, J. (1979). Retrodifferentiation and the fetal patterns of gene expression in cancer. *Adv. Cancer Res.*, **29**, 127.